# Role of Serial Polio Seroprevalence Studies in Guiding Implementation of the Polio Eradication Initiative in Kano, Nigeria: 2011–2014

**DOI:** 10.1093/infdis/jiv774

**Published:** 2016-04-02

**Authors:** Kehinde TemilolaOluwa Craig, Harish Verma, Zubairu Iliyasu, Pascal Mkanda, Kebba Touray, Ticha Johnson, Abdullahi Walla, Richard Banda, Sisay G. Tegegne, Yared G. Yehualashet, Bashir Abba, Amina Ahmad-Shehu, Marina Takane, Roland W. Sutter, Peter Nsubuga, Ado J. G. Muhammad, Rui G. Vaz

**Affiliations:** 1 World Health Organization, Country Representative Office; 2 National Primary Health Care Development Agency, Abuja; 3 Aminu Kano Teaching Hospital; 4 Bayero University, Kano, Nigeria; 5 World Health Organization, Geneva, Switzerland; 6 World Health Organization, Regional Office for Africa, Brazzaville, Congo; 7 Global Public Health Solutions, Atlanta, Georgia

**Keywords:** seroprevalence, poliomyelitis, Kano, Nigeria, DOPV

## Abstract

***Background.*** Nigeria was one of 3 polio-endemic countries before it was de-listed in September 2015 by the World Health Organization, following interruption of transmission of the poliovirus. During 2011–2014, Nigeria conducted serial polio seroprevalence surveys (SPS) in Kano Metropolitan Area, comprising 8 local government areas (LGAs) in Kano that is considered very high risk (VHR) for polio, to monitor performance of the polio eradication program and guide the program in the adoption of innovative strategies.

***Methods.*** Study subjects who resided in any of the 8 local government areas of Kano Metropolitan Area and satisfied age criteria were recruited from patients at Murtala Mohammed Specialist Hospital (Kano) for 3 seroprevalence surveys. The same methods were used to conduct each survey.

***Results.*** The 2011 study showed seroprevalence values of 81%, 75%, and 73% for poliovirus types 1, 2, and 3, respectively, among infants aged 6–9 months age. Among children aged 36–47 months, seroprevalence values were greater (91%, 87%, and 85% for poliovirus types 1, 2, and 3, respectively).

In 2013, the results showed that the seroprevalence was unexpectedly low among infants aged 6–9 months, remained high among children aged 36–47 months, and increased minimally among children aged 5–9 years and those aged 10–14 years. The baseline seroprevalence among infants aged 6–9 months in 2014 was better than that in 2013.

***Conclusions.*** The results from the polio seroprevalence surveys conducted in Kano Metropolitan Area in 2011, 2013, and 2014 served to assess the trends in immunity and program performance, as well as to guide the program, leading to various interventions being implemented with good effect, as evidenced by the reduction of poliovirus circulation in Kano.

In 1988, the World Health Assembly resolved to eradicate poliomyelitis by 2000 [1]. Subsequently, the Global Polio Eradication Initiative (GPEI) was able to reduce the number of polio-endemic countries and wild poliovirus (WPV) cases from 125 and 350 000, respectively, in 1988 to 3 and 416, respectively, by 2013 [2]. Indigenous transmission of WPV types 2 and 3 (WPV2 and WPV3) has since been interrupted globally [3, 4].

Nigeria was one of 3 remaining polio-endemic countries in the world, alongside Afghanistan and Pakistan [5], but on 25 September 2015, following the historical interruption of transmission of WPV for 14 months, the World Health Organization (WHO) director general, Dr Margaret Chan, on behalf of the WHO, delisted Nigeria as a polio-endemic country after 17 years of the country's polio eradication effort.

However, until 2012, Nigeria was the only country with transmission of all 3 poliovirus serotypes: WPV1, WPV3, and circulating vaccine-derived poliovirus type 2 (cVDPV2) [6], despite the conduct of multiple supplementary immunization activities (SIAs) and the use of more-immunogenic polio vaccines [7]. This indicated a substantial immunity gap, which led the country to determine how well children were protected against polio and where the remaining gaps driving persistent transmission were.

Based on the recommendations in the GPEI 2010–2012 strategic plan [8] on the conduct of polio seroprevalence surveys (SPS), the Nigerian government, with GPEI partners, decided to implement polio SPS in the high-risk states of the country. The objective was to estimate the level of population immunity, measure the programmatic progress through repeat surveys, and provide valuable insight for development of innovative strategies to interrupt poliovirus transmission.

Kano State has long been considered the epicenter of polio transmission in Nigeria and as one of the world's poliovirus sanctuaries [9, 10]. The state continues to be at high risk, such that even with substantial reduction in the recorded number of polio cases in Nigeria in 2014, it accounted for 5 of 6 WPV1 cases and 10 of 30 cVDPV2 cases in the country. By the recent joint WHO, Centers for Disease Control and Prevention (CDC), and Global Goods classification, 20 of the 38 local government areas (LGAs; 53%) at very high-risk (VHR) for polio transmission are from Kano State. All 8 urban LGAs of Kano Metropolitan Area (KMA)—Kano Municipal, Fagge, Nassarawa, Dala, Gwale, Tarauni, Ungogo, and Kumbotso—fall into this category. Based on this, Kano State, particularly the KMA (Figure 1), was the natural first choice for the survey.

**Figure 1.**
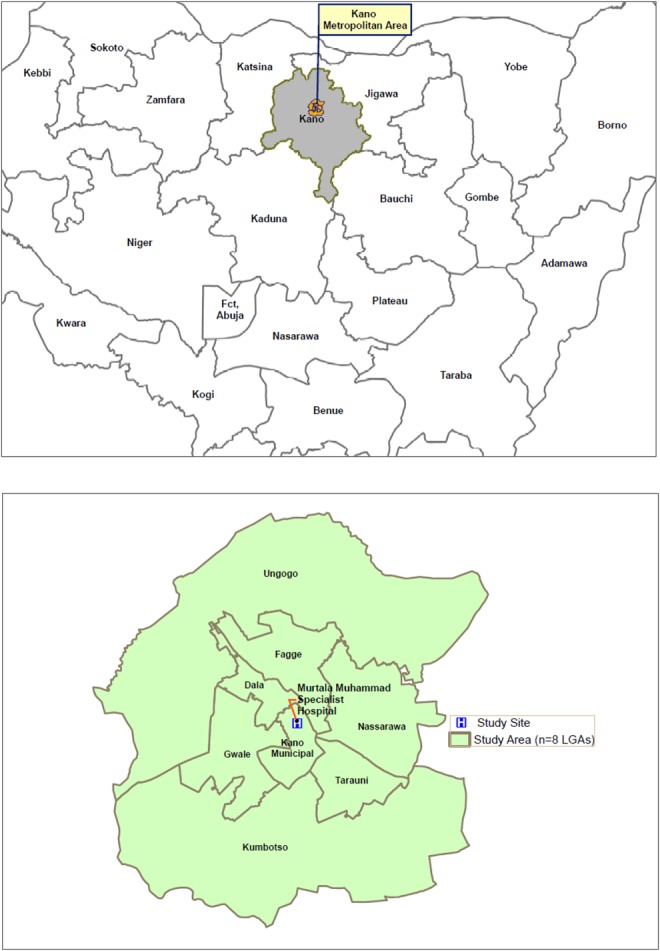
A map of the study area, Kano Metropolitan Area, in Kano State, northern Nigeria. Abbreviation: LGA, local government area.

Previous work has been done to measure and report the seroprevalence of polio serotypes among different age groups. Giwa et al reported that seroprevalence to poliovirus serotypes, although higher than values found in previous studies done in Nigeria, was lower than in the developed world [11]. This was corroborated by Iliyasu et al, who submitted that seroprevalence levels found in their survey, specific to Kano, were much lower than in corresponding serosurveys in Egypt and India [12, 13] and are insufficient to interrupt poliovirus transmission [14].

We present results of serial health facility–based polio seroprevalence surveys conducted in the 8 urban LGAs of KMA in Kano State, focusing on infants aged 6–9 months and comparing their seroprevalence levels in 2011, 2013, and 2014 to show how findings of these SPS have been used to review the polio eradication program in Nigeria and to subsequently develop innovative strategies for polio eradication.

## METHODS

### The Study

We began the initial survey with site assessment visits to the potential institutions, meeting potential investigators and deciding on participating institutions, the study team, and a feasible operational plan. The protocol received approval from the institutional review board of Aminu Kano Teaching Hospital (Kano) and the WHO Ethical Review Committee (Geneva, Switzerland).

A health facility–based cross-sectional design was chosen for operational ease, using the busy pediatric outpatient department of Murtala Mohammed Specialist Hospital (Kano). Study subjects fulfilling the 2 criteria of age and LGA of residence among those attending the pediatric outpatient department of Murtala Mohammed Specialist Hospital were recruited for the study after receiving care for the conditions that led to their hospital visit. The research physician then screened further for eligibility, obtained informed consent from the parent (as well as assent from children aged 10–14 years), and administered a questionnaire. Finally, blood samples were collected from the subjects. At the end of the survey, the samples were shipped to the CDC laboratory (Atlanta, Georgia), where neutralizing antibodies to the poliovirus serotypes were assessed following a standard protocol [15]. Children with detectable antibody levels at a ≥1:8 dilution were considered seropositive for that specific poliovirus serotype.

The studies were managed by combined teams of the WHO (headquarters and country office), the Kano State Ministry of Health, Aminu Kano Teaching Hospital, and the National Primary Health Care Development Agency.

### Dissemination of Results and Identification of Interventions

The results from these SPS were compiled and disseminated to the country's National Polio Emergency Operations Center and to the Expert Review Committee for Polio Eradication and Routine Immunization (ERC) for consideration and recommendations.

At the 24th ERC meeting, in 2012, following review of the results of the first Kano SPS of 2011, a recommendation was made to extend the SPS to other high-risk states, as well as to other age groups [16]. This led to the 2013/2014 SPS in Sokoto and Kebbi states and the repeat survey in Kano in 2013, involving 4 age groups (6–9 months, 36–47 months, 5–9 years, and 10–14 years), compared with the 2 age groups (6–9 months and 36–47 months) in the 2011 Kano SPS.

At the National Polio Emergency Operations Center, the Emergency Operations Center Strategic Group, headed by the WHO, reviewed the results, especially the low seropositivity recorded in infants aged 6–9 months, and various strategies were adopted and prioritized for implementation.

Furthermore, to assess the impact of additional doses from the various immunization plus days (IPDs) on the low immunity profile of this age group, a third SPS was conducted in October 2014 in Kano, this time in 2 age groups, 6–9 months and 19–22 months (ie, the children from the cohort aged 6–9 months at the time of the previous SPS, in 2013).

In 2014, for the first time, all study participants were geocoded and mapped, and the analysis subsequently conducted provided valuable support to programmatic interventions. For example, Figure 4 shows the location of seronegative and zero-dose subjects at the ward level within the LGAs. This geospatial analysis confirmed where there was a high density of zero-dose children during routine immunization (RI) activities and SIAs, as well as a high number of seronegative subjects. With these data, the program could see where the gaps in vaccination coverage were and, in turn, where there was a need to further review ongoing strategies.

To improve the program, some specific strategies were adopted and implemented, while some were scaled up. With respect to SIAs, accelerated inactivated polio vaccine (IPV) introduction in campaigns was started in Kano in December 2014, with enumeration of residents in targeted areas before implementation. Also health camps, which had earlier been suspended for security reasons, were reintroduced, and combined vaccination with oral polio vaccine (OPV) and IPV in KMA LGAs in Kano was conducted in the March 2015 IPDs. All children aged 0–59 months received OPV, and children aged 3–59 months also received an IPV dose.

The directly observed OPV (DOPV) strategy was scaled up to include KMA LGAs. This strategy was instituted to ensure vaccination of children in noncompliant households and other homes where children were being finger-marked by youths or mothers before vaccination teams arrived, as well as households in which vaccination teams colluded with mothers to finger-mark children without vaccination. Furthermore, monitoring through lot quality assurance surveys (LQAS) was modified to estimate the number of OPV doses received by children aged <5 years.

The RI intensification project was established in February 2015 in LGAs with cVDPV2 and low population immunity, and IPV was introduced into the RI schedule. In addition, the 2014 SPS findings were used as a high-powered advocacy tool to encourage buy-in by the Kano State government into the new strategies that had hitherto been conducted in other parts of the state but had been resisted in KMA.

## RESULTS

The polio SPS in 2011 revealed poliovirus seroprevalence values in the group aged 6–9 months to be 81%, 75%, and 73% for poliovirus types 1, 2, and 3, respectively, with higher values (91%, 87%, and 85%, respectively) in the group aged 36–47 months.

**Figure 2.**
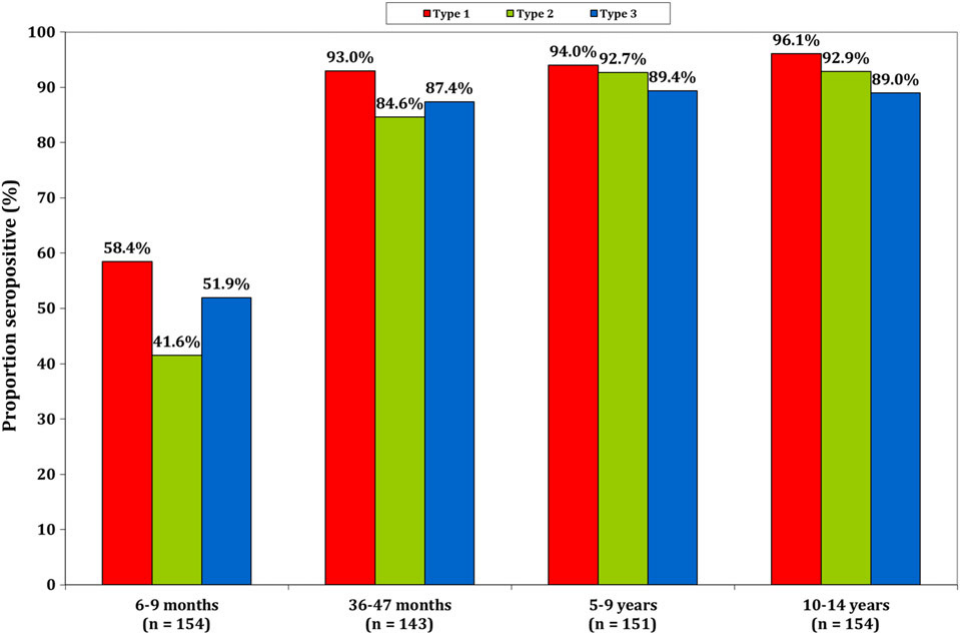
Poliovirus seroprevalence among children, by age group, in Kano, Nigeria, during 2013.

Seropositivity in the 2013 SPS, which was conducted in 4 age groups, was low among infants aged 6–9 months and high among children aged 36–47 months, with slightly greater frequencies of seropositivity among children aged 5–9 years and those aged 10–14 years (Figure 2). Among the children aged 6–9 months, the seroprevalence was 58% (95% confidence interval [CI], 51%–66%) to serotype 1, 42% (95% CI, 34%–50%) to serotype 2, and 52% (95% CI, 44%–60%) to serotype 3, whereas among children aged 36–47 months, the seroprevalence was 93% (95% CI, 88%–96%) to serotype 1, 85% (95% CI, 78%–90%) to serotype 2, and 87% (95% CI, 81%–92%) to serotype 3. The seroprevalence levels increased only marginally in the groups aged 5–9 years and 10–14 years.

**Figure 3.**
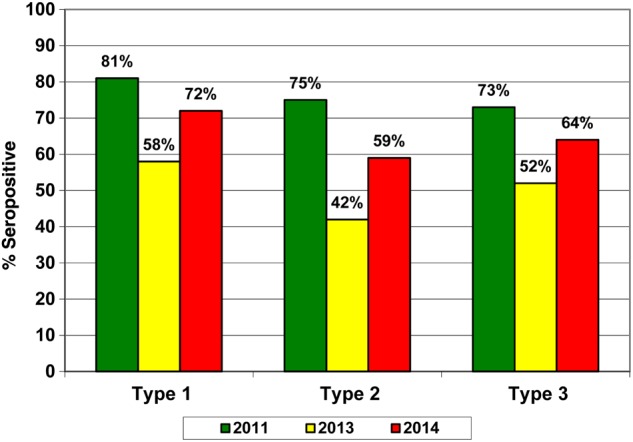
Seroprevalence, by poliovirus serotype, among infants aged 6–9 months in Kano Metropolitan Area, Kano State, northern Nigeria, during 2011, 2013, and 2014.

Figure 3 shows a comparison of the seroprevalence for all 3 serotypes among infants aged 6–9 months. The highest levels were in 2011 and for serotype 1. Seroprevalence for serotype 2 remained low across the 3 surveys, at <60%.

There was a positive correlation between the combined number of RI and SIA OPV doses received and seroprevalence levels for all serotypes in the group aged 6–9 months in 2014. There was an increasing trend in seroprevalence levels for all serotypes as the number of RI doses increased, with the highest seroprevalence in children who received the greatest number of doses and the lowest in those who received no immunization. These differences in all groups were statistically significant (*P* < .0001). However, with the SIA doses, this correlation was only true for serotypes 1 and 3 (Table 1) because SIA doses were mostly of bivalent vaccine (bOPV).

**Table 1.** Seroprevalence of Poliovirus Among Infants Aged 6–9 Months, by Number of Oral Polio Vaccine (OPV) Doses Received—Kano State, Nigeria, 2014

Dose Type, No.	Children, No.	Seroprevalence, No. (%)					
		PV1	*P* Value	PV2	*P* Value	PV3	*P* Value
RI OPV doses, no.			<.001		<.001		<.001
0	41	18 (43.9)		11 (26.8)		13 (31.7)	
1	13	9 (69.2)		6 (46.2)		10 (76.9)	
2	18	16 (88.9)		11 (61.1)		13 (72.2)	
3	20	14 (70.0)		13 (65.0)		13 (65.0)	
4	89	74 (83.2)		66 (74.2)		68 (76.4)	
SIA OPV doses, no.			.036		.705		.013
0	28	19 (67.9)		15 (53.6)		13 (46.4)	
1–3	57	37 (64.9)		35 (61.4)		38 (66.7)	
4–6	67	51 (76.1)		42 (62.7)		43 (64.2)	
≥7	26	23 (88.5)		15 (57.7)		22 (84.6)	
Total doses, no.			<.001		.002		<.001
0	6	2 (33.3)		2 (33.3)		0 (0.0)	
1–3	36	20 (55.6)		15 (41.7)		16 (44.4)	
4–6	55	38 (69.1)		32 (58.2)		38 (69.1)	
≥7	84	71 (84.5)		58 (69.1)		63 (75.0)	

Abbreviations: PV1, poliovirus type 1; PV2, poliovirus type 2; PV3, poliovirus type 3; RI, routine immunization; SIA, supplementary immunization activity.

**Figure 4.**
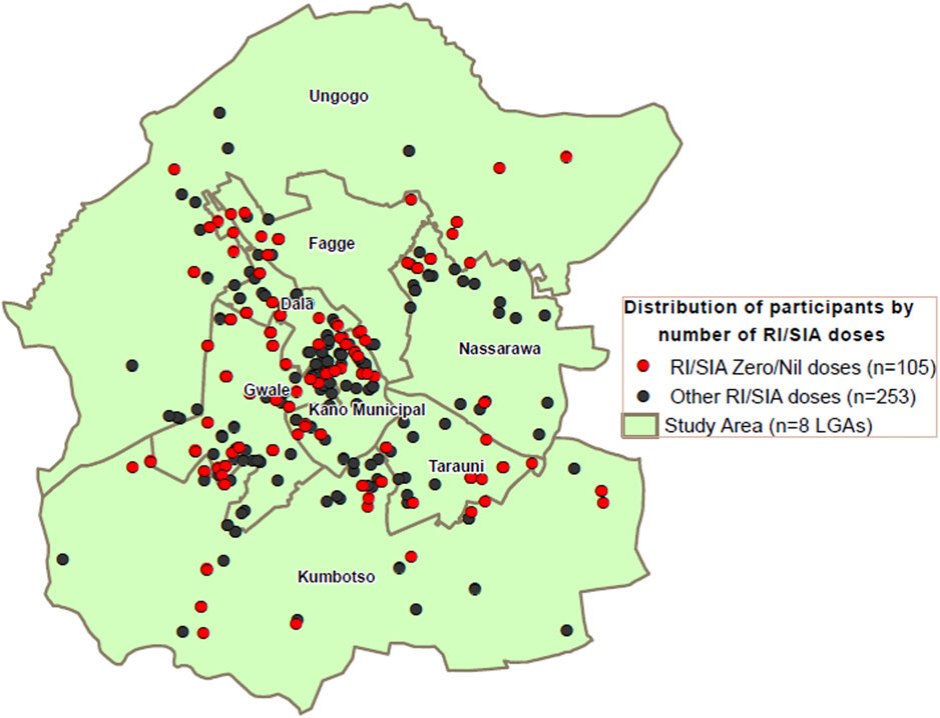
Geographic information systems analysis of the distribution of individuals who received 0 or ≥1 dose of polio vaccine during routine immunization (RI) or supplementary immunization activities (SIAs) in 2014, based on polio seroprevalence surveys. Abbreviation: LGA, local government area.

Geographic information systems analyses in Figure 4 show concentrated areas of zero-dose children from RI activities and/or SIAs as revealed by the survey. These areas and the high number of seronegative subjects indicate where the gaps in immunization coverage are.

**Figure 5.**
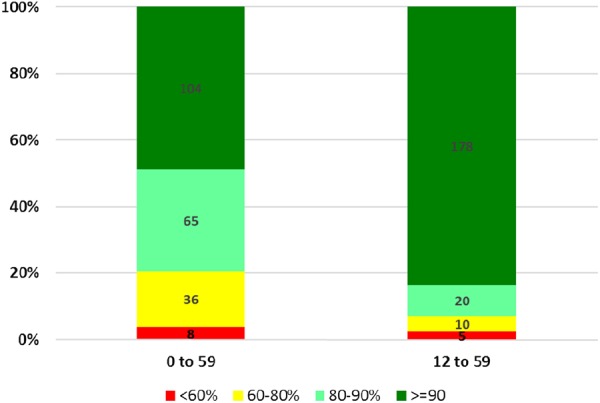
Modified data from a lot quality assurance survey of 213 local government areas in December 2014, showing the percentages of children, by age group, who had received ≥3 doses of oral polio vaccine since birth.

Dose profiling based on modification of the December 2014 LQAS data was performed to indicate the proportion of children aged 0–59 months and the subset aged 12–59 months who had received ≥3 OPV doses since birth (Figure 5). Analysis revealed that fewer children aged 0–59 months group had received ≥3 OPV doses since birth, compared with the group aged 12–59 months, by the end of the December 2014 IPD round.

**Figure 6.**
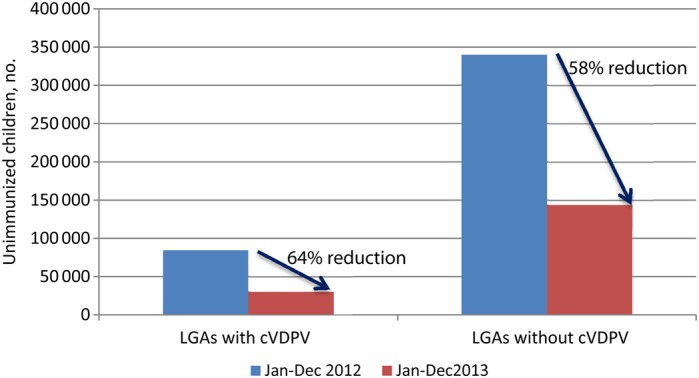
Results from the routine immunization intensification project. Comparison of the numbers of unimmunized children in 8 local government areas (LGAs) with circulating vaccine-derived poliovirus (cVDPV) and 36 LGAs without cVDPV in Kano State during January–December 2012 and January–December 2013.

The RI intensification project showed a 64% reduction in the number of unimmunized children in 8 Kano LGAs where cVDPV is circulating, compared with 58% among 36 Kano LGAs where cVDPV is not circulating (Figure 6). There was also a marked reduction in number of unimmunized children overall, from approximately 43 000 during January–December 2013 to approximately 15 000 during January–December 2014.

## DISCUSSION

We found that the proportion of infants aged 6–9 months who were seropositive to the 3 poliovirus serotypes remained consistently low in the 3 facility-based polio SPS that were conducted in KMA in the same health facility, thereby identifying a major gap that contributed to the persistent poliovirus transmission in Kano.

The marked drop in seroprevalence for all 3 serotypes in the group aged 6–9 months between 2011 and 2013 brought into focus the quality of IPD operations and the need to devise new strategies to reach the younger children, especially newborn babies and infants, most of whom would be with their mothers and therefore be in the category of “child absent” during IPDs. The evidence provided by the study yielded an increase in resources for demand-creation activities targeting mothers and caregivers of children in this age group.

An improvement in seroprevalence of 14% for type 1, 7% for type 2, and 12% for type 3 was recorded in 2014, compared with 2013. We also found that the seroprevalence for serotype 2 was low, at <60%, during the 3 surveys; seroprevalence was highest in 2011 but decreased in 2013 and 2014.

We also observed a clear correlation between seroprevalence and the number of OPV doses received by the study subjects, giving us evidence that the suboptimal seroprevalence we recorded arose because children were not being reached with repeated doses of OPV as required. Our findings were in agreement with those of Giwa et al, who submitted that immunization was the single most important determinant of seropositivity to poliovirus serotypes [11].

In RI activities, there is an increase in seroprevalence for all serotypes, since trivalent OPV (tOPV) is used. For SIAs, there is correlation only with serotypes 1 and 3, owing to continuous administration of bOPV during SIAs in Kano. This has provided evidence supporting the conduct of more SIAs, using tOPV. Comparison of the combined doses from RI activities and SIAs with seroprevalence data showed the trend clearly. This positive correlation between the number of OPV doses and the prevalence (≥90%) of higher antibody titers among children who received >7 OPV doses clearly addresses concerns about receipt of too many OPV doses that are often expressed by health professionals and noncompliant parents [17].

The geospatial analyses, which were included in the 2014 SPS for the first time, proved to be a valuable tool because they validated the findings of areas of high density of zero-dose children during RI activities and/or SIAs, as well as the high number of seronegative subjects. These findings encouraged the program to target innovative strategies on the younger age group, to conduct more tOPV SIAs, and to remunerate IPD vaccination teams and community informants with 1000 Nigerian naira (US$5) for every confirmed case of acute flaccid paralysis found. The directly observed OPV (DOPV) strategy was implemented in very high-risk and very very high-risk areas (61 of 69), which included the KMA.

IPV has proved successful in protecting children in areas where the prevalence of immunity is low, with an efficacy of >90%, as seen in studies conducted in Ivory Coast in 1993 [18]. Nigeria started using IPV in Borno, Yobe, and 12 LGAs of Kano State beginning in December 2014, and IPV was introduced in KMA LGAs as one of the strategies for improvement, during the March 2015 IPDs. According to the Kano Emergency Operations Center Report, a total of 956 499 and 861 174 children were immunized with OPV and IPV. In February 2015, IPV was introduced into the routine immunization program of Nigeria.

The dose profiling done using the LQAS data (Figure 5) showed that fewer children aged 0–59 months received ≥3 OPV doses, compared with the group aged 12–59 months, during the IPD round of December 2014. This difference is due to the population aged <1 year, which is generally underimmunized, as corroborated by results of our seroprevalence surveys for KMA. Based on LQAS data from January 2015, the proportions of 0-dose children and underimmunized children, by LGA, were estimated and the location of 0-dose children among those aged 0–35 months and 36–59 months were mapped for prioritization.

The RI intensification project in LGAs with cVDPV2 and low population immunity, which was established to tackle the persistently low seroprevalence to poliovirus type 2, resulted in an 80% reduction of unimmunized children over 6 months of implementation.

Our study was hospital based, but this limitation may have resulted in an overestimation of seroprevalence of antibody against poliovirus, as the children who are not reached by immunization activities may be less likely to visit hospitals. The results, although not generalizable, have been sufficient for the program to define innovative strategies to reach more children in targeted age groups, and this has contributed in the interruption of WPV circulation in Kano and the country as a whole.

The results of the serial SPS conducted in KMA, a very very high-risk area of Kano City, have helped bring focus to the quality of SIAs and to develop new strategies targeting the group aged 6–9 months, in which the seroprevalence was lowest. These innovative strategies have contributed to the reduction of WPV transmission in Kano and elsewhere in the country. Nigeria has not recorded any WPV cases in the year, and the country has since been delisted as a polio-endemic country, having interrupted polio transmission! As we recommended, another SPS was conducted in Kano in October 2015 to fully appreciate the impact of all these interventions.
